# Comparative Analysis of Click Chemistry Mediated Activity-Based Protein Profiling in Cell Lysates

**DOI:** 10.3390/molecules181012599

**Published:** 2013-10-11

**Authors:** Yinliang Yang, Xiaomeng Yang, Steven H. L. Verhelst

**Affiliations:** Lehrstuhl für Chemie der Biopolymere, Technische Universität München, Weihenstephaner Berg 3, 85354 Freising, Germany; E-Mails: yinliangyang@gmail.com (Y.Y.); xiaomeng@hotmail.de (X.Y.)

**Keywords:** activity-based probes, cathepsins, click chemistry, proteases, protein modification

## Abstract

Activity-based protein profiling uses chemical probes that covalently attach to active enzyme targets. Probes with conventional tags have disadvantages, such as limited cell permeability or steric hindrance around the reactive group. A tandem labeling strategy with click chemistry is now widely used to study enzyme targets *in situ* and *in vivo*. Herein, the probes are reacted in live cells, whereas the ensuing detection by click chemistry takes place in cell lysates. We here make a comparison of the efficiency of the activity-based tandem labeling strategy by using Cu(I)-catalyzed and strain-promoted click chemistry, different ligands and different lysis conditions.

## 1. Introduction

**Figure 1:**
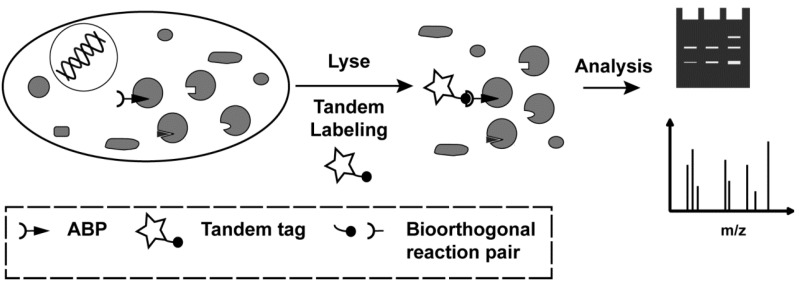
Schematic representation of tandem ABPP as site specific and activity-based technique to covalently tag active enzymes *in* *situ* and *in vivo*. A small molecule ABP equipped with one partner of a bioorthogonal reaction pair forms a covalent bond with active site residues of a subset of enzymes in a cell. After lysis, tandem labeling, such as click chemistry, introduces a tagging moiety on the enzyme-ABP complex. Depending on the tag, different analysis methods can be used, such as in-gel scanning, or affinity enrichment with mass spectrometry detection.

Within chemical biology, site specific protein modification by covalent small molecule probes is a powerful and often used technique to interrogate biomolecular interactions and protein function. The introduction of covalent small molecule probes can be based on different types of chemistries: probes may be incorporated by the use of exogenous or endogenous enzymes [1,2], or they can contain an intrinsic reactivity such as an electrophile or photocrosslinker that by itself forms a covalent bond to the target proteins [3,4,5]. Generally, the probes can be dramatically adjusted in their selectivity by a combination of the reactive groups and additional structural elements that interact with the target proteins [6]. Activity-based protein profiling (ABPP) is a particularly interesting method, since it is able to address the functional state of a protein, *i.e.* active or inactive [3,4]. This is especially important for enzymes, which are dynamically regulated after translation. ABPP makes use of small molecules termed activity-based probes (ABPs) that are able to covalently tag active enzymes. ABPs generally consist of three elements: (1) a tag, (2) a reactive group that covalently reacts with a residue in the active site of the target enzymes, often in a mechanism-based way, and (3) a spacer with optional recognition elements that can induce selectivity. The method of detection of the modified proteins depends on the nature of the tag, such as a biotin or a fluorophore [7]. Most tags are relatively bulky compared with the small molecule probe, which influences the cell permeability and may prevent that the reactive group enters the active site. To circumvent these problems, two-step (tandem) labeling strategies using bioorthogonal reaction partners have been increasingly applied [8]. The 1,3-dipolar cycloaddition between azides and alkynes, commonly referred to as click chemistry [9,10] has especially become popular. Since its introduction in tandem ABPP by Speers and Cravatt [11,12], azide or alkyne ‘mini-tags’ are now very common in activity-based probes (ABPs) and their applications *in situ* or *in vivo* (Figure 1) [13]. Several studies on the efficiency of the click reaction have been reported. Some have used fluorogenic click substrates, others metabolically labeled proteins, cell lysates or even whole cells or organisms for evaluation [14,15,16,17]. These studies have, for example, found out that some ligands for stabilization of the Cu(I) species lead to higher rates of cycloaddition than others.

Unless detection *in vivo* or in intact cells is desired, such as in imaging, most tandem labeling in ABPP takes place in cell lysates. In this workflow, lysates, whole cells or organisms are first incubated with an ABP. Only after cells or tissues are lysed, click chemistry is performed and subsequent analysis takes place (Figure 1). One recent study has compared the usage of copper-free tandem labeling in ABPP (Staudinger ligation and strain-promoted click chemistry) [18]. However, not only the click reagents, but also the constituents of the lysate may have an influence on the tandem labeling efficiency. Therefore, we here report a comparative study on the efficiency of tandem labeling in ABPP using different click chemistry conditions and different lysis methods.

## 2. Results and Discussion

### 2.1. Activity-Based Probes and Fluorescent Tags

**Figure 2:**
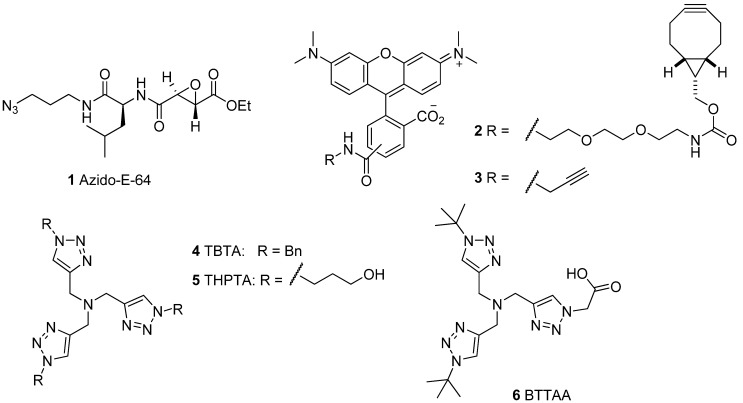
The molecules used in this study: the cell permeable ABP azido-E-64 (**1**), two alkyne tags for click chemistry (**2** and **3**), and three ligands for Cu(I)-catalyzed click chemistry.

In order to test the efficiency of click chemistry in ABPP, we used the cell permeable ABP azido-E-64 as a model probe [19] (**1**; Figure 2). It is based on the natural product E-64, which is a covalent inhibitor for cysteine proteases of the papain family [20]. Probe **1** is cell permeable and covalently modifies its target proteases at the active site cysteine. For in-gel detection of the ABP-protease complex we use fluorescent tags, since fluorescence provides high sensitivity and is easily detected by scanning wet gels. Hence, we selected two alkyne-containing fluorescent tags: the terminal alkyne **3** for Cu(I)-catalyzed click chemistry, and the bicyclononyne (BCN) derivative **2** for Cu(I)-free, strain-promoted click chemistry. The latter compound was synthesized in a one step procedure from a commercially available BCN derivative [21] with a short PEG linker and carboxy-tetramethylrhodamine succinimide ester.

Because Cu(I) is relatively instable in solution, click chemistry has benefited from ligands that chelate and stabilize Cu(I). The first reported ligand is the widely used tris[(1-benzyl-1H-1,2,3-triazol-4-yl)methyl]amine (**4**; TBTA; Figure 2) [22]. This ligand was also the first ligand in tandem ABPP with click chemistry [11]. Other ligands such as THPTA (**5**) [14] and BTTAA (**6**) [15] have been developed to improve properties like water solubility. We here use these three ligands to compare their performance in the tandem ABPP.

### 2.2. Tandem Labeling Experiments

**Figure 3:**
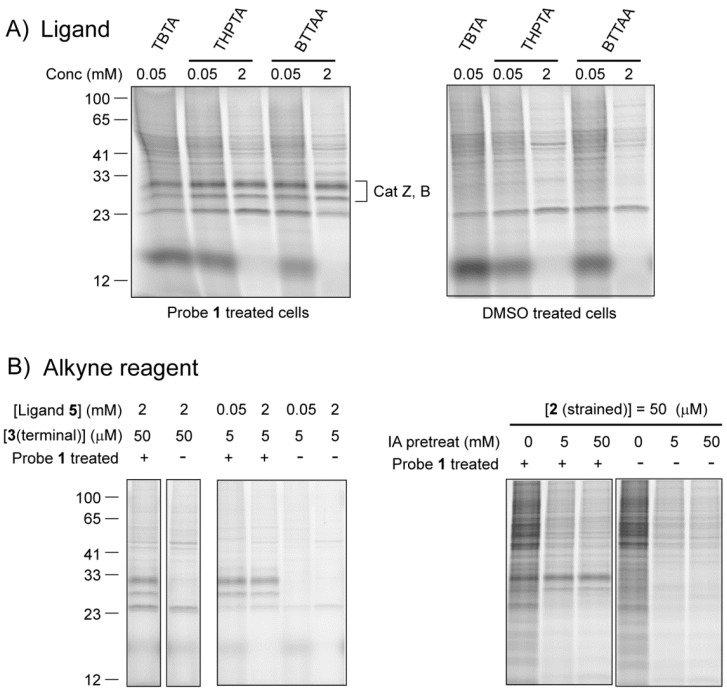
(**A**) Comparison of the three different Cu(I) ligands for click chemistry. Cells were treated for 1 h with 5 μM of probe **1**, washed and lysed with 1% NP-40 in 100 mM sodium phosphate pH 7.4. Lysates (diluted to 1 mg/mL total protein, 0.1% NP-40 final concentration) were incubated for 1 h with 1 mM CuSO_4_, 1 mM sodium ascorbate, 50 μM or 2 mM ligand and 50 μM of the terminal alkyne tag **3**. (**B**) Comparison of Cu(I)-catalyzed and strain promoted click chemistry in lysates. Samples were treated similar as under (a) with THPTA as a ligand, but with different concentrations of the alkyne reagent. For strain-promoted click chemistry, only reagent **2** was added. Right panel: samples were pre-treated for 30 min with the indicated concentration of iodoacetamide (IA), then treated with strained alkyne **2** (5 μM).

To obtain a proteome with a small number of selectively azide-labeled proteins, we incubated the macrophage cell line RAW264.7 with 5 μM azido-E-64 (**1**) for 1 h. The main targets of azido-E-64 in this cell line are cathepsin Z and B in the molecular weight range of 26-30 kDa [23] (Figure 3a). After washing of the cells to remove free ABP, we performed lysis of the cells under different conditions (see Section 2.2.2). As a negative control, DMSO vehicle treated cells were taken and subjected to the same lysis conditions. The resulting proteomes were subsequently used in the click chemistry labeling procedures.

#### 2.2.1. The Influence of Click Chemistry Reagents

We first compared ligands **4-6** in the tandem tagging of the azide-labeled cathepsins with Cu(I)-catalyzed click chemistry. Due to its limited solubility, TBTA (**4**) has been used as a ligand in ABPP at substoichiometric amounts with regard to the Cu^+^ concentration (50 μM:1 mM) [11,12]. THPTA (**5**) and BTTAA (**6**) and can be used at much higher concentrations [14,15]. We here used **5** and **6** in either low (50 μM) or high (2 mM) concentration with a fixed [Cu^+^] of 1 mM. Although BTTAA (**6**) has shown higher kinetics of click chemistry in aqueous environment [15], we here do not observe a substantial difference in the intensity of the fluorescenly tagged cathepsins at 26–30 kDa (Figure 3A; Figure S1). TBTA (**4**) performs only slightly worse than the other two (far left lane). The majority of the higher molecular weight bands as well as a band at approximately 22 kDa are a result of background labeling independent of the presence of the azide-E-64 probe, as can be seen in the samples from DMSO treated control cells (Figure 3A; right panel). A higher ligand:copper ratio led to an overall lower amount of background. The intensity of target labeling, however, is not affected by the ligand:copper ratio.

In order to reduce the background, we decided to take a look at the influence of the alkyne reagent. A tenfold lower concentration of terminal alkyne **3** gave rise to a substantially lower background with similar intensties of the cathepsin bands (Figure 3B, left panel). Hence, a low concentration of alkyne tag seems beneficial for a detection with better signal to background ratio. In sharp contrast, strain-promoted click chemistry, which is a popular alternative to the copper catalyzed version, gave rise to very high non-specific staining (Figure 3B, right panel). This is most likely due to a thiol-yne reaction between cysteine containing proteins and the strained alkyne [15,24]. Lowering the concentration of the strained alkyne **2** to 5 μM reduced the non-specific signals, but did not result in the clear cathepsin signals as observed with the Cu(I)-catalyzed click reaction (Figure S2). Blocking the free cysteines prior to strain-promoted click chemistry has been reported to lower the thiol-yne background labeling [25]. Upon pre-treatment of the lysate with iodoacetamide, we indeed observed a lower amount of non-specific staining, and obtained clearer signals of target cathepsins (Figure 3B; right panel). Altogether, the Cu(I)-catalyzed click reaction outperforms the strain-promoted one in the detection of labeled targets in cell lysates. These optimized conditions with an azide probe and a terminal alkyne tag show virtually identical labeling patterns compared with an alkyne probe and an azide tag (Figure S3), which was reported to give lower background [12].

#### 2.2.2. The Influence of Lysis Conditions

**Figure 4:**
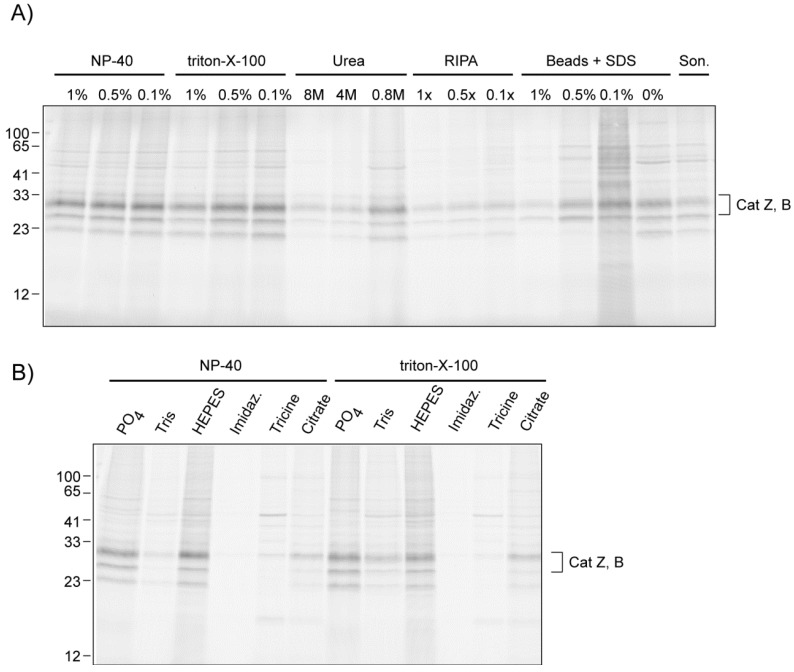
(**A**) Comparison of different lysis conditions on the tandem ABPP of cathepsins by Cu(I)-catalyzed click chemistry. All buffers except RIPA contained 100 mM sodium phosphate. SDS was added post lysis with glass beads. All protein concentrations were adjusted to 1 mg/mL. Click chemistry was performed for 1 h with 5 μM of alkyne **3**, 2 mM THPTA, 1 mM CuSO_4_, and 1 mM sodium ascorbate. (**B**) Comparison of different buffering salts (all at pH 7.4; final detergent concentration 0.1%) on the tandem ABPP of cathepsins.

There are a wide variety of methods to make lysates from mammalian cells. Most make use of detergents to disrupt the cell membrane, while others use sonication or glass bead disruption. We have here used different lysis conditions to investigate the effect of buffer additives like detergents on the click chemistry efficiency (Figure 4A). High concentrations (1%) of strong, ionic detergents like SDS or deoxycholate had a negative effect on the efficiency, as did the usage of high concentrations of urea. Dilution led to signals with higher intensity. Mild, non-ionic detergents did not show this dramatic effect. Detergent-free cell disruption using glass beads or sonication also gave low band intensities, possibly caused by worse solubilization of the cathepsin targets. Addition of SDS to these lysates also had a negative effect on the click chemistry.

We also investigated different buffering agents used in cell lysis buffers: sodium phosphate, Tris, HEPES, imidazole, tricine and citrate (Figure 4B). Both sodium phosphate and HEPES were compatible with the tandem ABPP labeling. Tris showed a decreased click chemistry efficiency, as it can act as an inhibitory ligand for the Cu(I) species [14]. A similar decrease was observed for imidazole, tricine and citrate buffers.

## 3. Experimental

### 3.1. Materials

CuSO_4_, propargylamine hydrochloride, diisopropylethylamine and bicyclo[6.1.0]non-4-yn-9-ylmethyloxycarbonyl]-1,8-diamino-3,6-dioxaoctane were purchased from Sigma-Aldrich (Schnelldorf, Germany), NP40 substitute, Triton-X100, SDS, DMSO, HEPES, Tris, imidazole, tricine, citric acid and sodium deoxycholate were obtained from AppliChem (Darmstadt, Germany), sodium dihydrogenphosphate was purchased from Carl Roth (Karlsruhe, Germany), and 5- and 6-carboxytetramethylrhodamine, succinimidyl ester was received from Life Technologies (Darmstadt, Germany). TBTA [22], THPTA [14], azido-E-64 [19] were synthesized as reported. BTTAA [15] was a gift of the laboratory of Prof. Dr. Stephan Sieber (Technical University Munich, Department of Chemistry).

TAMRA-BCN conjugate **2**: BCN-PEG-amine (3.59 mg, 0.011 mmol) was dissolved in DMSO (400 µL). 5- and 6-carboxytetramethylrhodamine, succinimidyl ester (4.86 mg, 0.009 mmol) and DIEA (1.93 µL, 0.011 mmol) were added and the solution was incubated for 4 h. The final product was purified by reversed phase HPLC to give a red compound (3.7 mg; yield 56%). ESI-HRMS: [M+H]^+^ calculated for C_42_H_48_N_4_O_8_ 737.3472, found 737.3483.

TAMRA-propargylamine **3**: 5- and 6-carboxytetramethylrhodamine, succinimidyl ester (3.2 mg, 0.006 mmol) was dissolved in DMSO (400 µl). Propargylamine hydrochloride (1.2 mg, 0.012 mmol) and DIEA (4 µL, 0.023 mmol) were added and the solution was incubated overnight. The final product was purified by reversed phase HPLC to give a red compound (1.88 mg; yield 67%). ESI-HRMS: [M+H]^+^ calculated for C_28_H_25_N_3_O_4_ 468.1845, found 468.1878.

### 3.2. Cell Culture and Generation of Lysates

RAW 264.7 cells were cultured in DMEM medium containing 10% heat-inactivated fetal bovine serum (FBS), 100 units/mL penicillin, and 100 μg/mL streptomycin and maintained in a humidified 37 °C incubator with 5% CO_2_. Cells with nearly 90% confluence were incubated for 1 h at 37 °C with fresh medium containing alkyne-E-64 (from a 5 mM DMSO stock solution (1000×); final concentration of alkyne-E64: 5 µM; final DMSO concentration: 0.1%). Next, cells were washed with PBS (2×), harvested by using a cell scraper, collected by centrifugation, and lysed by the following methods: resuspension in 1% triton-X100 or 1% NP-40 in either 50 mM Tris pH 7.4, 50 mM HEPES pH 7.4, 100 mM sodium phosphate, pH 7.4, 50 mM tricine, pH 7.4, 50 mM imidazole, pH 7.4, or 50 mM citric acid, pH 7.4, and incubation on ice for 30 min (with vortexing every 10 minutes). The same was done for RIPA buffer (50 mM Tris, 150 mM NaCl, 1% triton-X100, 1% sodium deoxycholate, 0.1% SDS, pH 7.4). Incubation with 8 M urea was followed by sonication. For lysates made with class beads, an equal volume of glass beads was added to the cell pellet together with detergent-free buffer. The mixture was vortexed for 30 s, followed by incubation on ice for 1 min. This process was repeated 3 times. For all lysis conditions, cell debris and unlysed cells were removed by centrifugation (15,000 rpm) for 20 minutes. The supernatant was snap-frozen in liquid nitrogen and stored at −80 °C until further usage. Protein concentrations were determined by Bradford or DC protein assay (Bio-Rad, Munich, Germany).

### 3.3. Bioorthogonal Labeling Reactions

Azido-E-64-labeled proteome was diluted to 1 mg/mL total protein concentration with the appropriate buffer to obtain final detergent or additive concentrations as indicated. The click chemistry was carried out in a volume of 100 µL For Cu(I)-catalyzed tagging, the reaction mixture was incubated at room temperature for 1 h after the addition of 1µL of TAMRA-propargylamine **3** (0.5 mM or 5 mM stock in DMSO), 1 µL of ligand (5 mM and 200 mM stocks in DMSO), 2 µL of sodium ascorbate (50 mM stock in H_2_O) and 2 µL of CuSO_4_ (50 mM stock in H_2_O). Note that the solutions of sodium ascorbate and CuSO_4_ were freshly made. For strain promoted click chemistry, the reaction mixture was incubated at room temperature for 1h after the addition of 1 µL of **2** (0.5 mM or 5 mM stock in DMSO). For alkylation of the free thiols and subsequent labeling, the lysate was first incubated with iodoacetamide (5 or 50 mM) for 30 min followed by strain promoted click chemistry as described above. All reactions were stopped by adding 400 µL of cold acetone and the mixture was stored at −20 °C for 30 min. After centrifugation (15,000 rpm, 4 °C, 20 min) the supernatant was removed and protein pellets were dissolved in 1x sample buffer (132 µL). Thirty µL of this solution was separated by 15% SDS-PAGE. Gels were directly scanned on a Typhoon Trio+ (PMT = 600). Gels were stained with Coomassie to check for equal protein loading.

## 4. Conclusions

Click chemistry is an important tool in bioorthogonal labeling of azide-functionalized biomolecules. Using tandem ABPP, we have evaluated the labeling efficiency in cell lysates as a function of different click chemistry conditions and different lysis methods. We were able to make several observations: (1) The nature of the ligand to stabilize the Cu(I) species did not have a substantial influence on the tandem labeling procedure in ABPP. Although differences in click chemistry kinetics have been reported for ligands **4**-**6**, the 1 h incubation time and the conditions that were used in the tandem ABPP may have caused these differences to be less pronounced. Hence, we recommend the usage of **5** which is commercially available and can be used at a higher concentration than ligand **4**, resuling in a lower background labeling. (2) Strain promoted click chemistry with fluorescent tag **2** gave rise to a high amount of background, consistent with earlier observations [18]. Although strained cyclooctynes have been successfully applied [26], for example in conjugations of purified biomolecules or in imaging of cell surface glycans, the usage in cell lysates is not advised. Lowering of the concentration of terminal alkyne tag **3** led to a decrease in background labeling, (3) The use of Tris, imidazole, tricine and citrate buffer gave lower click efficiencies than HEPES or phosphate buffer. The last two buffers are therefore preferred. (4) Strong, ionic detergents such as SDS and deoxycholate, influenced the tandem labeling in a negative way, as did the presence of high concentrations of urea. Thus, when possible, it is desirable to use mild, non-ionic detergents and dilute the lysate before click chemistry to lower the final detergent concentration.

## Supplementary Materials

Supplementary materials can be accessed at: http://www.mdpi.com/1420-3049/18/10/12599/s1.
