# Supplementary material for: Comparative Analysis of Click Chemistry Mediated Activity-Based Protein Profiling in Cell Lysates
Source: Molecules. 2013 Oct 11;18(10):12599–608. doi: 10.3390/molecules181012599 (PMC6270401; doi:10.3390/molecules181012599)

Supplementary Materials

Figure S1. Comparison of the three different Cu(I) ligands for click chemistry. Related to Figure 3a; Triton-X-100 used for cell lysis. Cells were treated for 1 h with 5 μM of probe **1**, washed and lysed with 1% Triton-X-100 in 100 mM sodium phosphate pH 7.4. Lysates (diluted to 1 mg/mL total protein and 0.1% Triton-X-100 final concentration) were incubated for 1 h with 1 mM $CuSO_4$, 1 mM sodium ascorbate, 50 μM or 2 mM ligand and 50 μM of the terminal alkyne tag **3**. Lysates from DMSO treated cells were used as background controls.

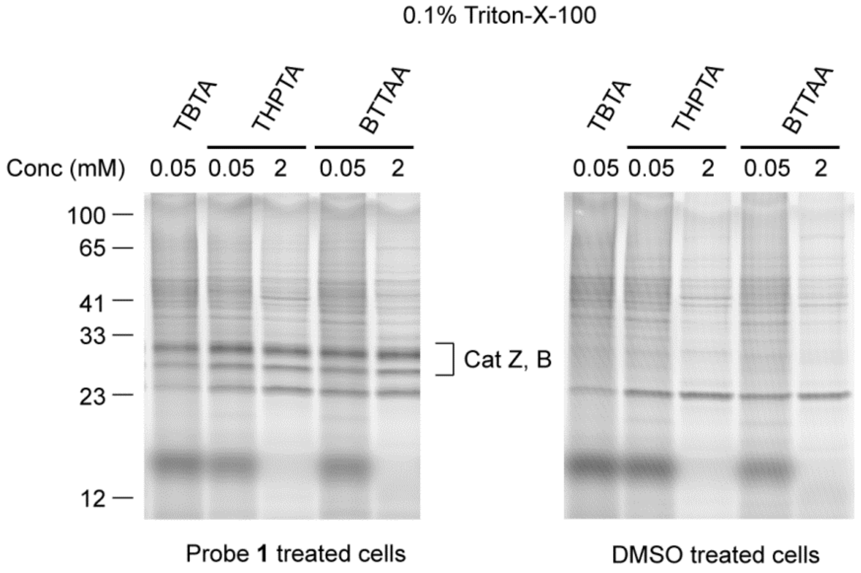

Figure S2. Comparison of Cu(I)-catalyzed and strain promoted click chemistry in lysates. Related to Figure 3b; 5 μM alkyne tag used. Cells treated with probe **1** or DMSO were lysed with 1% NP-40 in 100 mM phosphate buffer (pH 7.4). Lysates (diluted to 1 mg/mL total protein and 0.1% NP-40 final concentration) were then subjected to click chemistry. For strain-promoted click chemistry, only reagent **2** was added and incubated for 1 h. For copper-catalyzed click chemistry (right two lanes), samples were incubated with 1 mM $CuSO_4$, 1 mM sodium ascorbate, 2 mM THPTA and 5 μM of reagent **3**.

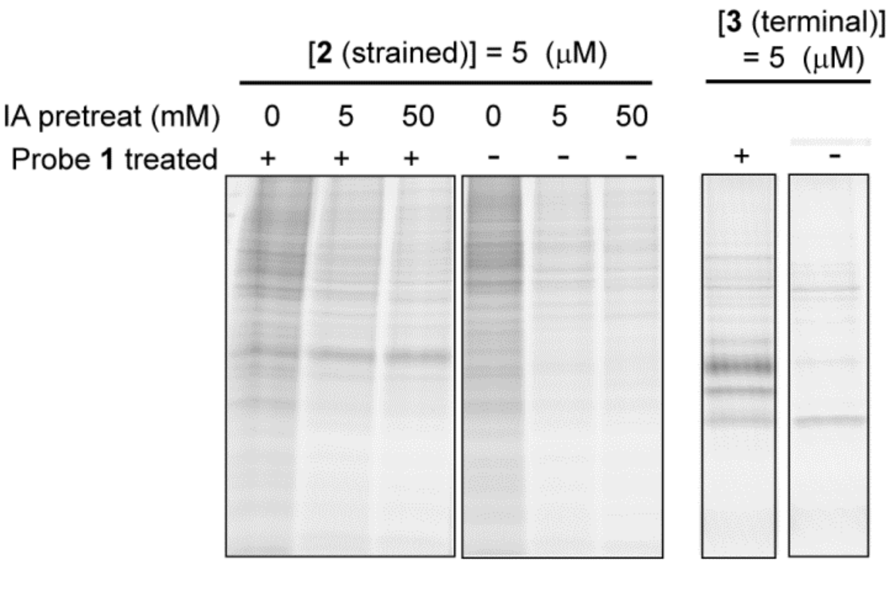

Figure S3. Comparison of the Cu(I) catalyzed click chemistry with azide and alkyne probes. Cells were treated for 1 h with 5 µM of probe **1**, probe **7** or DMSO, washed and lysed with 1% NP-40 in 100 mM sodium phosphate (pH 7.4). Lysates (diluted to 1 mg/mL total protein; final concentration of NP-40 of 0.1%) were incubated for 1 h with 1 mM $CuSO_4$, 1 mM sodium ascorbate, 50 µM or 2 mM ligand and 50 µM of the terminal alkyne tag **3** or azide tag **8**.

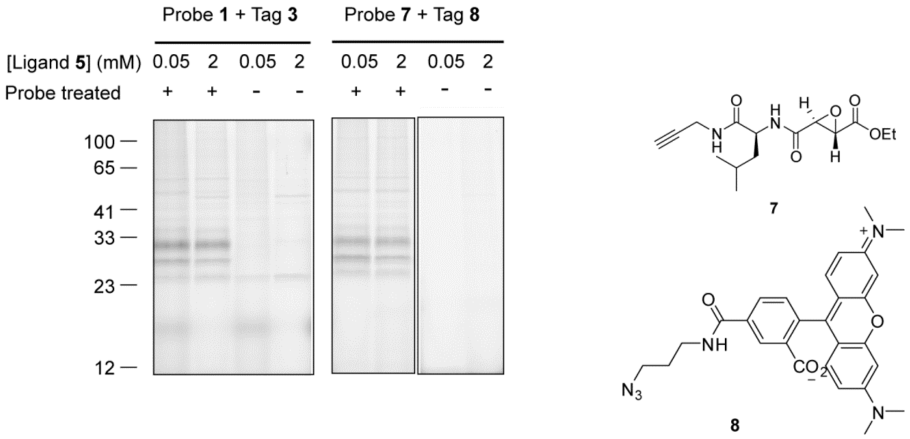

Compound **2**

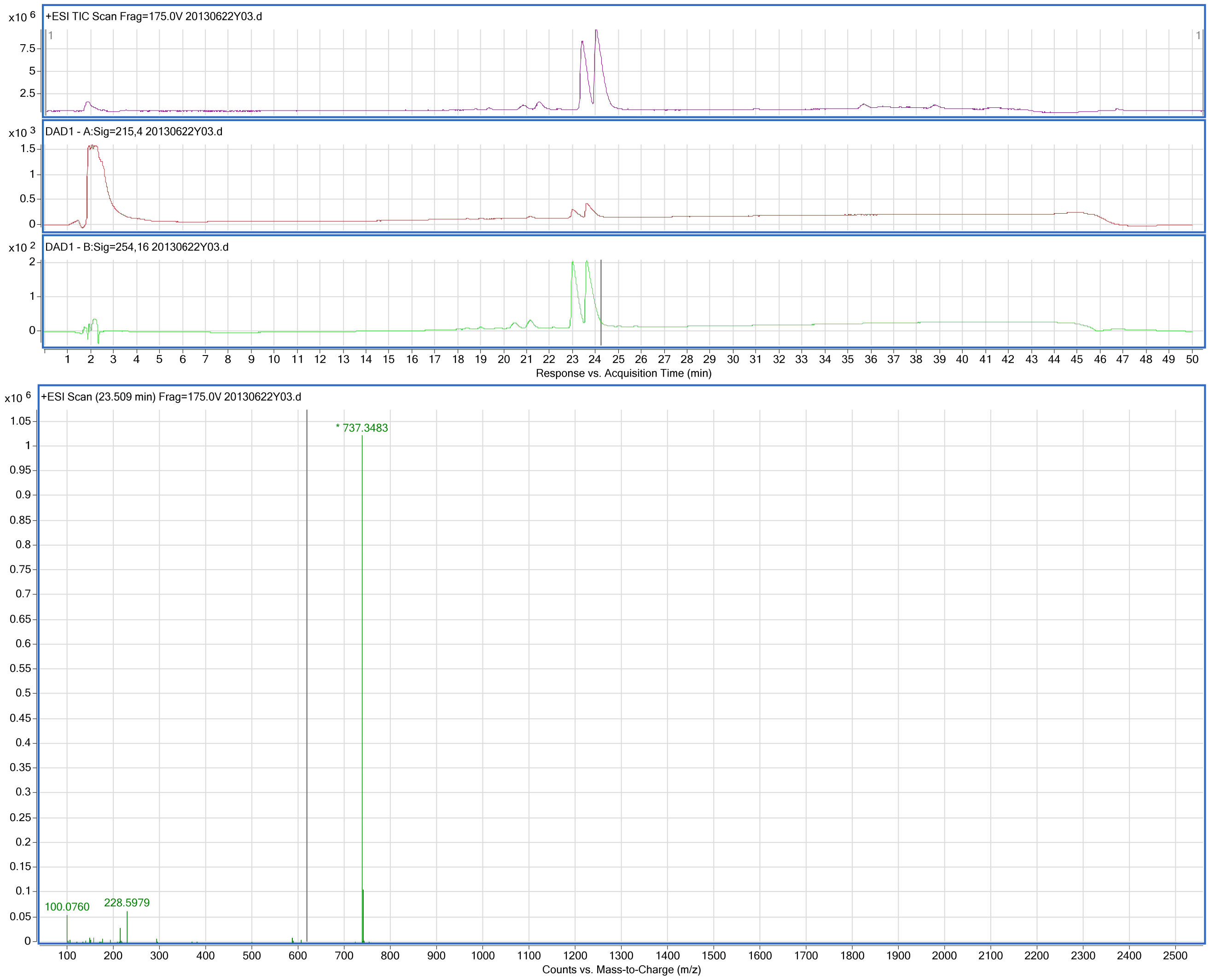

Figure S3. *Cont.*

Compound **3**

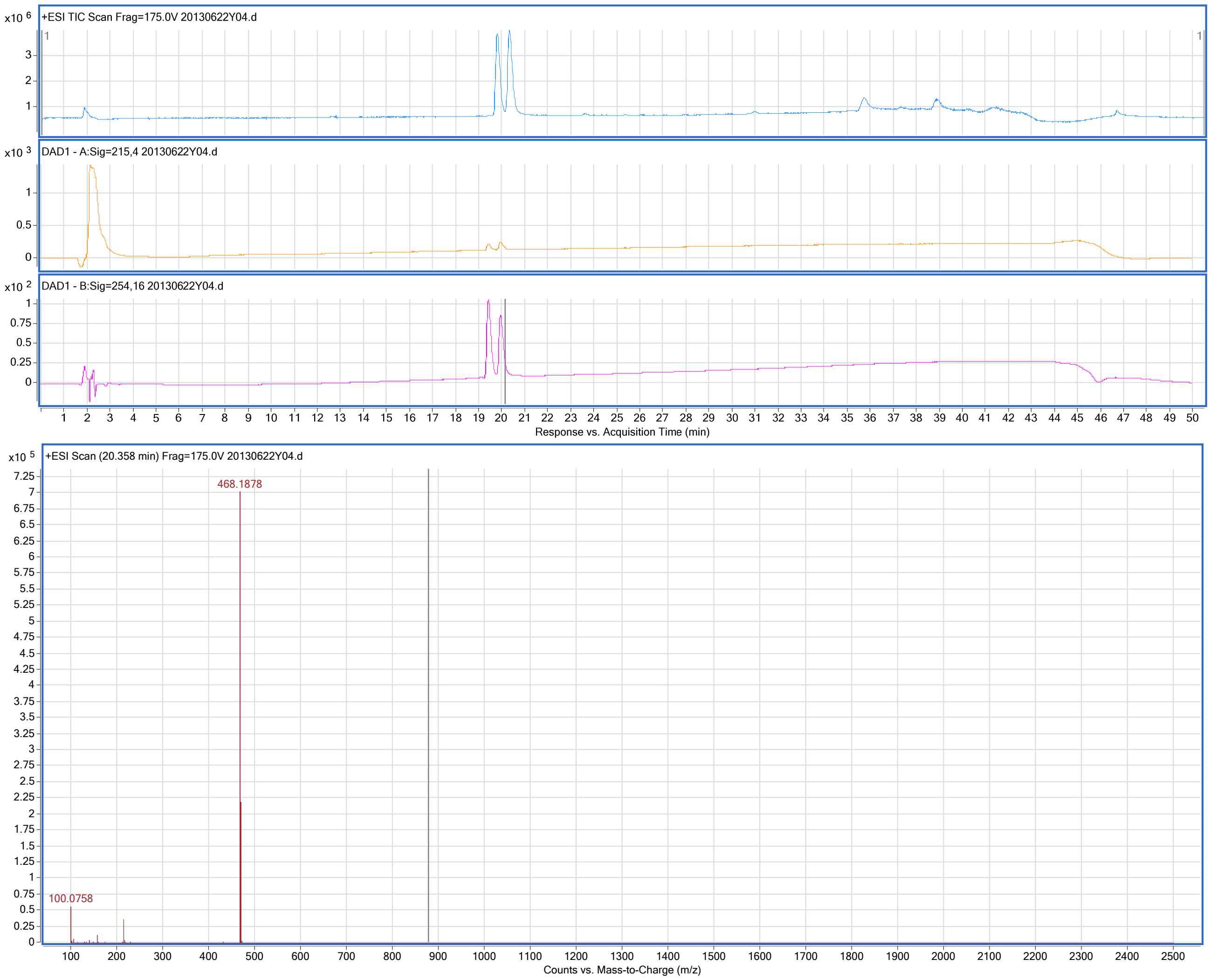

Supplement: Supplementary file 1 [file molecules-18-12599-s001.pdf]
